# Supplementary figures and images for: Molecular Analysis of Indole and Skatole Decomposition Metabolism in Acinetobacter piscicola p38 Utilizing Biochemical and Omics Approaches
Source: Microorganisms. 2024 Aug 29;12(9):1792. doi: 10.3390/microorganisms12091792 (PMC11434297; doi:10.3390/microorganisms12091792)

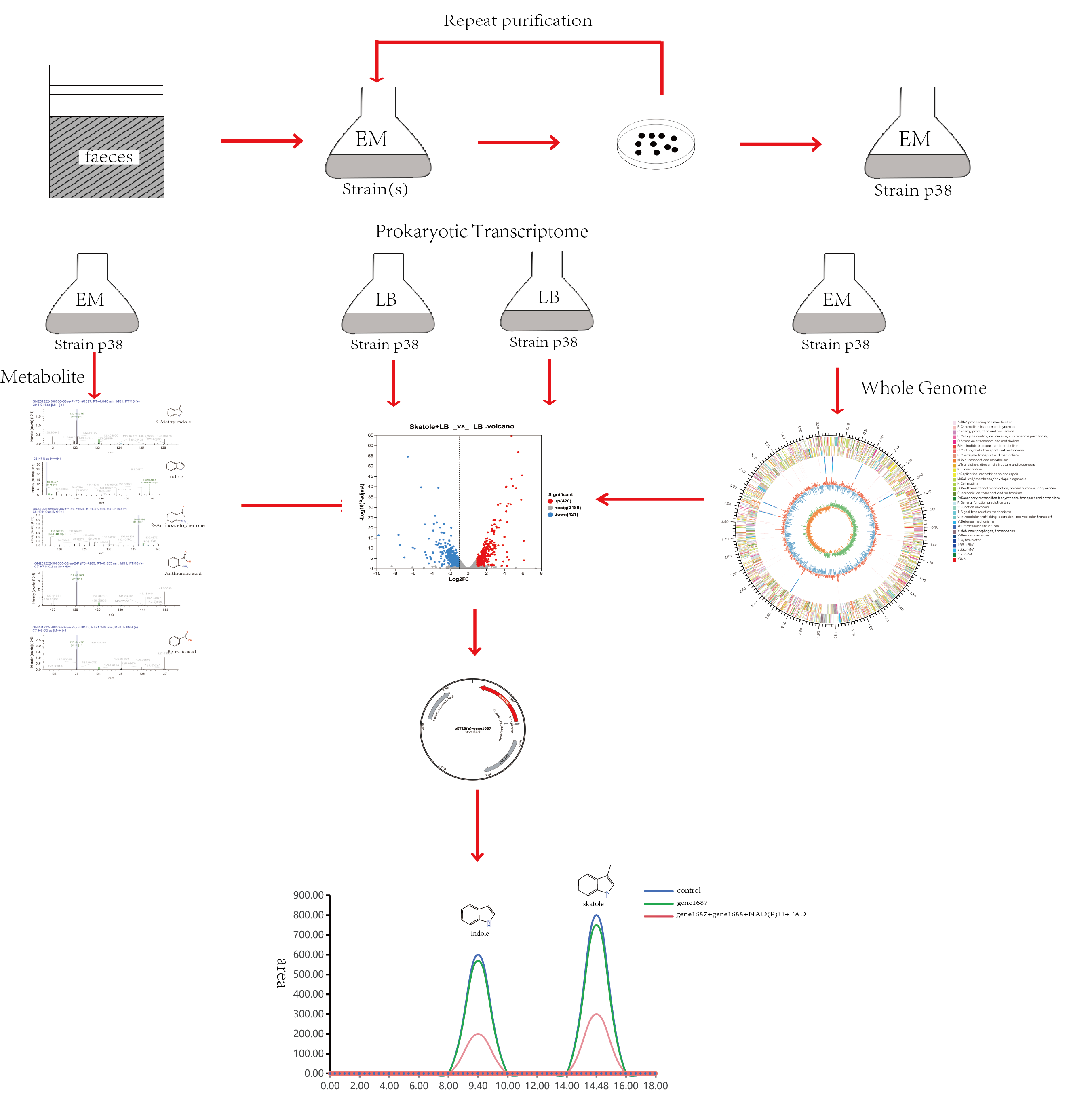

Supplement: Supplementary file 1 [file microorganisms-12-01792-s001.zip › Supplementary File/Graphical abstract.png]
